# Evaluation of the determinants of food security within the COVID-19 pandemic circumstances- a particular case of Shaanxi, China

**DOI:** 10.1186/s41256-021-00230-2

**Published:** 2021-12-01

**Authors:** Apurbo Sarkar, Wang Hongyu, Abdul Azim Jony, Jiban Chandro Das, Waqar Hussain Memon, Lu Qian

**Affiliations:** 1 College of Economics and Management, Northwest A&F University, Yangling, 712100 Shaanxi China; 2 School of International Education, Xidian University, Xian, 710071 Shaanxi China; 3 School of Mechanical Engineering, Nanjing University of Science and Technology, 200 Xiaoling Wei, Nanjing, 210014 China; 4 College of Economics and Management, Sher e-Bangla Agricultural University, Dhaka, 1207 Bangladesh

**Keywords:** Food security, Key-aspects, Production, Distribution, Structural equation modeling, Determinants

## Abstract

**Background:**

Agricultural food production and distribution industries may play a vital role in determining the current conditions of any country’s food security and sustainable development goals. This paper examined the determinants of food security within three distinct aspects (effective utilization of food, food availability, and food access) within the COVID-19 epidemic situation.

**Methods:**

The qualitative set-up of the study comprised with the identification of drivers by critical analysis of published papers and discussion held with some practitioners. The quantitative data used in this research were collected from a survey covering the agricultural food supply industry in China (Shaanxi Province). The survey was conducted from November to December 2020 and we mainly focus on three aspects of food security (effective utilization of food, food availability, and food access). The core analytical assumptions were made by employing exploratory factor analysis (EFA), confirmatory factor analysis (CFA), and structural equation modeling (SEM).

**Results:**

After analyzing the data collected from 257 agricultural food productions and distribution personnel along with the hypothesis testing, it found that the determinants of the effective utilization of food were positively related to the determinants of food access (β = 0.291, *p* = 0.029) and food availability (β = 0.298, *p* = 0.011), and the determinants of food availability were positively related to the food access determinants (β = 0.128, *p* = 0.002). The association and variance values between food availability and food access were 0.659 and 0.407; the association and variance values between for effective utilization of food and food availability aspects were 0.465 and 0.298, and between effective utilization of food and economy were 0.508 and 0.475.

**Conclusion:**

The study critically evaluated the interconnection among the crucial determinants within the banner of three dimensions, which will act as a major contribution to existing literature. This research will help the government and industry to develop policies and strategies for the successful implementation of all the associated determinants of food security in terms of the epidemic situation.

**Supplementary Information:**

The online version contains supplementary material available at 10.1186/s41256-021-00230-2.

## Introduction

Since the epidemic of COVID-19 spread out, some apparent changes in regional food systems and most other agricultural production factors like the supply of manure, fertilizers, energy, pesticides, seeds, and shortage of labor have become visible. As a result, these possible earning and transition losses are supposed to create intense pressure and threaten food security (FS) in several regions of the world. Sound production and supplies of agricultural food have a strong influence on mitigating global food and nutritional requirements and maintaining the smooth development goal of the United Nations (UN) [1, 2]. The World Food Program of the United Nations has claimed approximately 265 million people are at risk of severe food insecurity by the end of the year 2020, a rise from 135 million inhabitants before catastrophe [3]. As purchasers have now become limited and suppliers ignore collaborating with growers, the food producers often face significant losses from nutritious and perishable food products. Several nations and enterprises are currently imposing extra initiatives to secure agricultural production, distribution, and smooth transition. It helps the government combat the possible food scarcity and maintain adequate supplies of reasonably priced and nutritious meals within the COVID-19 pandemic circumstances. So, general customers and inhabitants can still reach and buy food considering the imposed constraints on travel and income losses [4].

Due to the flourishing agricultural and distribution sectors, China is continuously holding a leading position in the global food supply chain [5]. However, the recent outbreak of the novel coronavirus (COVID-19) has dramatically changed the global and local scenarios [6]. From the beginning of the COVID-19 outbreak, the Chinese government imposed a stringent lockdown policy. The effects of these epidemic situations are usually many, but two significant components of the food supply chain (production and distribution) are severely exaggerated by this cataphoric virus outbreak [7]. For maintaining effective lockdown, several measures and controls are imposed on people’s mobility. Transport of agricultural inputs is found limited and the supply of labor is decreased, which potentially could cause disruptions in the agriculture production sector. From producer to consumer, from local buyers to wholesale, and from cross-regional shipping to city consumption, almost every channel of the agricultural product supply chain has been interrupted [8]. Moreover, the decreasing demand for agricultural products arises from strict lockdown and sudden shutdown of restaurants, local markets, and caterers result in enormous proportions of unsellable seasonal perishable vegetables and fruits, which are backlogged and even unpicked in the farmland eventually [9]. If such devastating problems could not be appropriately addressed, farmers would not gain any profit from the current harvesting which would also be troublesome for them to reinvest in the next spring plantation, resulting in vast constraints in next season’s growth. According to Food and Agriculture Organization (FAO) [10], the cumulative impacts of the COVID-19 outbreak have put the whole world in threatening conditions of food security and nutritional deficiencies, whereas the conditions of the countries with a large population are most critical. In summary, it can be stated confidently that food security determinants should be measured accordingly and structurally to provide a clear overview due to these severe circumstances.

In order to provide a clear overview of every country's food security, the prime issues that should be evaluated are what determinants quantify the FS as a complete and integrated system. Furthermore, the determinants should be measured and structured with some statistical tools for better interpretation. In this article, FS is quantified with several determining factors and indicators, which are categorized into three main aspects (effective food utilization, food access, and food availability) for a more precise representation of the proposed model. Those determinants play an active role in quantifying the effective measures for securing sufficient and nutritious food. Due to the recent outbreak of COVID-19, those determinants are facing continuous alterations. Therefore, the evaluation, incorporation, and assessment of these determinants are the first priority for maintaining desirable FS.

However, within the last few years, there are several studies, which have measured FS [11, 12], COVID-19 and FS [13], FS and human mobility [14, 15], the impact of COVID-19 on food and nutrition security [16], food insecurity and COVID-19 [17], food systems and FS [18], the determinants of FS [19, 20] and so on. By a careful evaluation and summarization of the contribution and findings of those individual articles, this study proposed a framework for quantifying food security within the circumstances of COVID-19.

According to the Food and Agriculture organizations of the United Nations (FAO), the effective utilization of food largely depends on the uninterrupted supply and availability of different types of food [21, 22]. Therefore, if a sufficient amount of food and clean water could accessible, it could have impacted the proper utilization of food [23]. On the other hand, if any burdens arise that hinder the smooth access to food, the effective utilization of food may be interrupted [24, 25]. It is expected that many people will not have proper food utilization due to a lack of access to nutritious food, especially within the pandemic situation raised by Covid-19 [26]. It is apparent that the pandemic situation has also hindered the food supply, which creates a shortage of adequate food supply than the market demands [27, 28]. According to Naja and Hamadeh [29], if the availability and access of food is viable and smooth the effective utilization of food can be fosters. Based upon the above discussion, we have crafted hypotheses 1 and 2:

### **H1**

The “effective utilization of food” would be positively related to “food access.”

### **H2**

The “effective utilization of food” would be positively related to “food availability.”

Food availability and food access are considered as both sides of a coin [30]. In the simplest term, food availability is the situation where food is made to exist for consumption at local levels where local individuals or households can access their needed foods without striving [31]. According to the World Food Program (WFP), if any country could not ensure an adequate food supply, it will become problematic for the general people to access an adequate diet, which eventually hinders food security [32]. The demand and supply of food become more vulnerable within the current circumstances of COVID 19. Erokhin and Gao [33] found, the supply of food has been interrupted most due to the pandemic situation, which hindered access to food among the investigated 45 developing countries. By evaluating food availability and access in African-American communities, Odoms-Young et al. [34], food availability largely quantified food accessibility. It is apparent that if the availability of food could be established, the access of nutrition would be fostered [35]. Thus, it could be a legitimate argument that food availability may significantly influence food access. Based on this, the study has crafted hypotheses three as:

### **H3**

The “food availability” would be positively related to “food access.”

The critical evaluation of the reviewed studies revealed that most of the studies developed the determinants based on prior literature. Furthermore, a few authors have verified the determinants by using some robust statistical instrument. However, no investigator has constructed a framework of determinants that reflects the interconnection between the determinants. This article is designed to address this gap in the literature by developing a framework to quantify the determinants of FS based on CFA and SEM tactics. Moreover, a limited number of studies have been traced that can quantify food security within the context of the COVID-19 circumstance. Also, the investigation of the food production and distribution industry is also mostly ignored by previous studies. A well-structured framework and exploration of the relationship among the determinants of FS within the context of COVID-19 circumstances are the main innovation of this paper.

Based on the research hypothesis, the main purpose of the study was to explore the determinant of food security within three distinct aspects within COVID-19 epidemic situation. To further decompose the main objectives we have explored the three main research questions. First, what are the principle determinants of food security during the COVID-19 situation? Second, whether and how these determinants are interconnected to each other? How to structure the determinant of food security within COVID-19 situation?

## Methods

### Research design

**Fig. 1:**
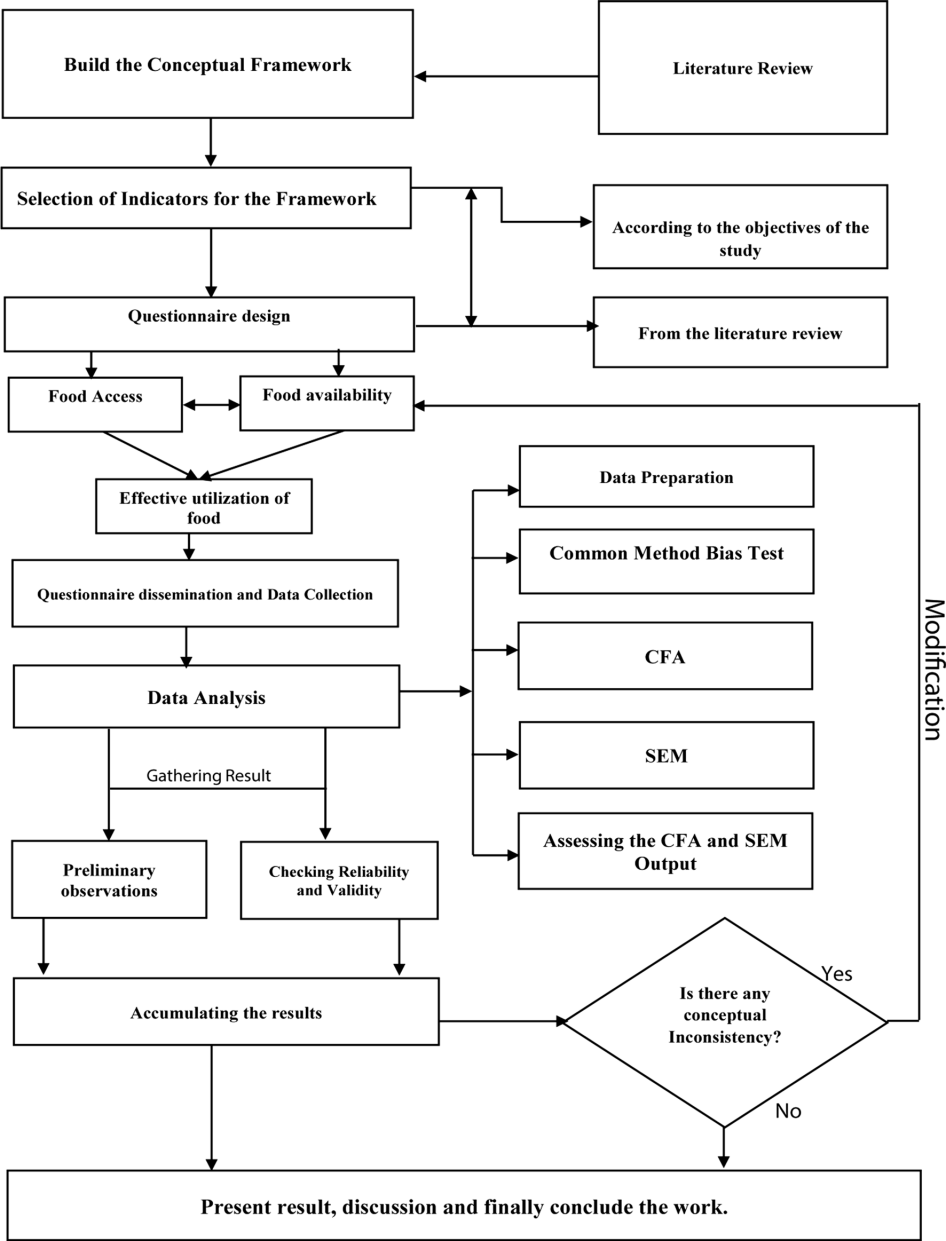
Steps of the current study. Adapted and modified from Sarkar et al. [37]

Both qualitative (anti-positivism) and quantitative (positivism) methodologies (approach) were utilized for fulfilling the objectives of this study. The basic steps and methodology were the indicators development, framework design, questionnaire development, and framework validation. The qualitative set-up of the study comprised with the identification of drivers by critical analysis of published papers and discussion held with some practitioners. We comprised 15 determinants from an in-depth literature investigation of several published peer-reviewed journal articles, books, regional and international reports, and some discussion with some professors and industry professionals at the beginning of the analysis. The quantitative aspects of the study were fostered by empirical data collection with a structured questionnaire. For developing and validating the model, the empirical data were collected from the agricultural food supply industry in China (Shaanxi Province). At the same time, the core analytical assumptions were made by employing exploratory factor analysis (EFA), confirmatory factor analysis (CFA), and structural equation modeling (SEM). The primary steps associated with the current research methodology are portrayed in Fig. 1. Different practitioners widely utilize these types of methodologies for a similar framework [36].

### Sample

The data used in this research were collected from a survey in Shaanxi province from November to December 2020. We used a multistage sampling procedure in selecting rural agriculture production and distribution personnel. First, we purposively chose seven counties within Shaanxi province. Second, within each county, we randomly selected 6–9 villages. Third, within each village, we randomly selected and interviewed one or two food production and distribution personnel. The sampling procedure resulted in a sample of 313 farm households from 23 villages (randomly selected).

### Variable and research model

The variables and indicator of the study have been extracted from the extensive review of the existing literature, which is generally used by similar studies. The list of the variables and indicator has been portrayed in Table 1. Seemingly, we have developed a model which is quantified by structural educational modelling by using the variables effective utilization of the food, food access and food availability.

**Table 1:** Selected indicator of FS of the study

Variables	Indicator	References
Food availability	Price of food	[27, 44–46]
	Production	[44, 47–49]
	Varieties of food	[27, 45, 50, 51]
	Proper distribution channel	[45, 47, 52, 53]
	Diverse & variety of retails options	[44, 47, 54]
Effective utilization of food	Purchase	[4, 24, 47, 52]
	Processing	[24, 48, 55, 56]
	Consumption	[27, 46, 50, 57]
	Changing strategies	[27, 54, 56, 57]
	Skill, knowledge and references	[45, 54, 55]
Food access	Physical access	[27, 45, 54]
	Financial access	[45, 46, 48, 55]
	Markets/infrastructures	[27, 45, 46, 52]
	Social supports	[7, 45, 50, 53]
	Timing	[44, 48, 50, 53]

As a multivariate tactic, partial least squares structural equation modeling (PLS-SEM) does not demand strict sample size and data normalization [38]; determining the appropriate sample size for producing an effective PLS-SEM model is a tricky question [39]. Hence, Hoyle [40] and Hair et al. [41] suggested that at least 100 observations could be the starting point for securing the estimation. Interestingly, Marcoulides and Saunders [42] addressed that the minimum sample size could be determined by the maximum number of arrows pointing at a latent variable. They suggested that if the maximum number of arrows counts as two, the minimum sample size should be 52. If the arrow number is five, then the sample should be 70, and if the arrow count is ten, then the minimum number of the sample should be comprised of at least 91 observations. With the help of a structured questionnaire, we gathered 257 responses with full information (those do not contain any missing information). Therefore, the final data set of 257 farmers has secured all the above-discussed parameters of minimum sample size.

### Survey instrument development

By focusing on the determinants portrays in Table 1, a structured survey questionnaire was developed. In the questionnaire, respondents were asked to assess how significant the determinants are for quantifying FS on five-point Likert scale feedback, where 2 denotes very less effect, and 10 denotes very substantial effect. This measure is often utilized within a similar domain since it explores the same interval between the specific calculation value and the interval scale and the measure is estimated and assisted with some other convenient quantitative analysis [36]. According to Voon et al. [43], for effective evaluation of the respondent’s personal judgment these types of questionnaires should be employed.

### Data collection

A structured questionnaire was disseminated via email, and We-Chat app with a detailed explanation of the objectives of the study, followed by the telephonic discussion that was held to find out the relationship among the identified determinants. In the initial stage of our survey, we encountered with low response rate. For addressing this, a follow-up mail and telephonic call were made to increase the response rate. From 313 polls, we finally got 257 usable fully filled questionnaires, which were further used for the interpretation of the SEM model.

### Statistical analysis

Data in the study has been analyzed into four stages. In the first part, all the respondents’ demographic information was gathered, and the second part consisted of the rating options for the determinants. A sample questionnaire are presented in Fig. A1 (please see Additional file 1).Second, the study explored the single factor biasness test to secure the viability if the data. Third, we employed Explanatory factor Analysis (EFA) for the framework proposition (see Additional file 1) and validation of the framework is confirmed by Confirmatory Factor Analysis (CFA) and Structural Educational Modeling (SEM) tactics. First, we utilized the EFA for evaluating the association among the latent and measured variables (see Additional file 1). Further, we utilized CFA for providing a substantial assessment of the construct’s uniformity. As Koufteros [58] suggested, EFA is not profound for confirming the theory or proposed framework. As an essential part of SEM tactics, CFA is a multivariate analytical framework that investigates the uni-dimensionality of the casual association among the latent and measured variables in a prior established model derived from theory [59]. This is crucial since weak correlations among conceptual factors and observed variables might cause inaccurate assumptions and create confusion for the interpretation of the relationship within the theoretical framework [58].

## Results

### Demographic analysis

A total of 257 responses with full information had been extracted from the survey (Table 2). Among them, 198 (around 76%) participants were males. Almost, 66% of them had at least high school degree and most of them belonged to the households with at least 3–5 members. Age ground of 36–40 found to be dominated (53%). The head of households were mostly 73% male and around 87% of the respondents were the main earning persons of the family.

**Table 2:** Demographic profile of the respondents

Attributes	Distribution	Frequency	Percent (%)
Gender	Male	198	76
	Female	59	24
Age	Under 25 years	18	7
	26–30 years	44	17
	31–35 years	46	18
	36–40 years	137	53
	Above 40 years	12	5
Family pattern	3 or lower member	66	26
	3–5 member	172	67
	6–8 member or above	17	7
Educational level	Primary School diploma or no education		15
	High School		66
	Bachelors-Higher		19
Household leader	Male	188	73
	Female	69	27
Earning characteristics	Main earner	223	87
	Subordinate	34	13

### Confirmatory factor analysis (CFA)

The observations had been transmitted to CFA by employing the AMOS (Moment Analysis Structure) statistical tool, and are presented in Table 3. As per the uniform and unregulated regression measurements, the findings of the observations are presented in Table S2 (see Additional file 1). Within the unstructured regression measurements, the values of the regression of a single item under all uniformed variables were specified, and the rest of the other variables were assumed. The regression values of “physical access, purchase, and price of food” were randomly specified. The correlation’s unstandardized value implied that the component raised the non-standardized value of the correlation against “the endogenous variable by 1”.

**Table 3:** A statistical representation of Confirmatory factor analysis

Determinants	Regression weights^a^			Regression weights^b^
	Estimate	SE	Critical ratio	
Price of food	1.000	–	–	0.748
Production	1.087	0.167	7.543	0.897
Varieties of food	0.796	0.145	5.654	0.588
Proper distribution channel	0.790	0.180	5.987	0.695
Diverse and variety of retails options	0.864	0.276	3.879	0.693
Purchase	1.000	–	–	0.478
Processing	1.656	0.379	3.976	0.678
Consumption	2.679	0.376	3.896	0.785
Changing strategies	2.235	0.689	4.659	0.887
Skill, knowledge and references	1.986	0.877	3.876	0.560
Physical access	1.000	–	–	0.795
Financial access	1.020	0.167	6.382	0.774
Markets/infrastructures	0.987	0.198	6.087	0.878
Social supports	0.778	0.147	5.290	0.609
Timing	0.870	0.368	3.867	0.689

^a^Unstandardized; ^b^Standardized. *p* < 0.001 (for all coefficients)

Structured regression values imply that if the latent factor increases by “1 standard deviation”, then the standard deviation of each factor is also increased by the structured regression values compared to that element [36]. Table S2 (see Additional file 1) confirms that for our framework the minimum value of standard error were 3.892, which was much higher than the minimum accepted weight (|2|) of the “critical ratio” for the variable estimate. While, |2| is often recommended for testing the significance at the 0.01 level, as stated by Huddleston-Casas et al. [60] and Mittal and Sangwan [36]. The framework might have standardized regression values of attributes ranging from 0 to 1. The higher the value is, the better the identified factors represent the latent factor. The findings showed that all the determinants hold values more than 0.6, which implies all the determinants reasonably signify the projected latent factors.

The measurement model was tested by using the MLE (maximum likelihood estimation), where 59 degrees of freedom (DF) and X^2^ (CMIN)-value of 152.81 were found. The ratio of X^2^ to DF came out to be 2.59, which was far below the highest suggested weight of 5 [36]. The *p* value of < 0.001 illustrated that the findings were statistically substantial, with more than 99% confidence level. The goodness of fit index value was 0.829, and the comparative fit index value was 0.853, which denoted that the CFA findings fit our proposed framework. The root means the square error of the approximation value was 0.09 (recommended value is close to zero), and the root mean square residual value was 0.07 (recommended value < 0.08). Moreover, the latent covariance generally ranges binary from “0” to “1”, by which “0” implies the considerations are distinct, and “1” implies that the overall considerations seem to be the same. The association and variance values for food availability and food access were 0.659 and 0.407; the association and variance values for effective utilization of food and food availability aspects were 0.465 and 0.298; and the association and variance values between effective utilization of food and economy were 0.508 and 0.475. Therefore, the conclusion was to verify the convergent validity and ready for thoroughly assessing with the structural framework to validate the ultimate framework of the determinants.

### Structural equation model

**Fig. 2:**
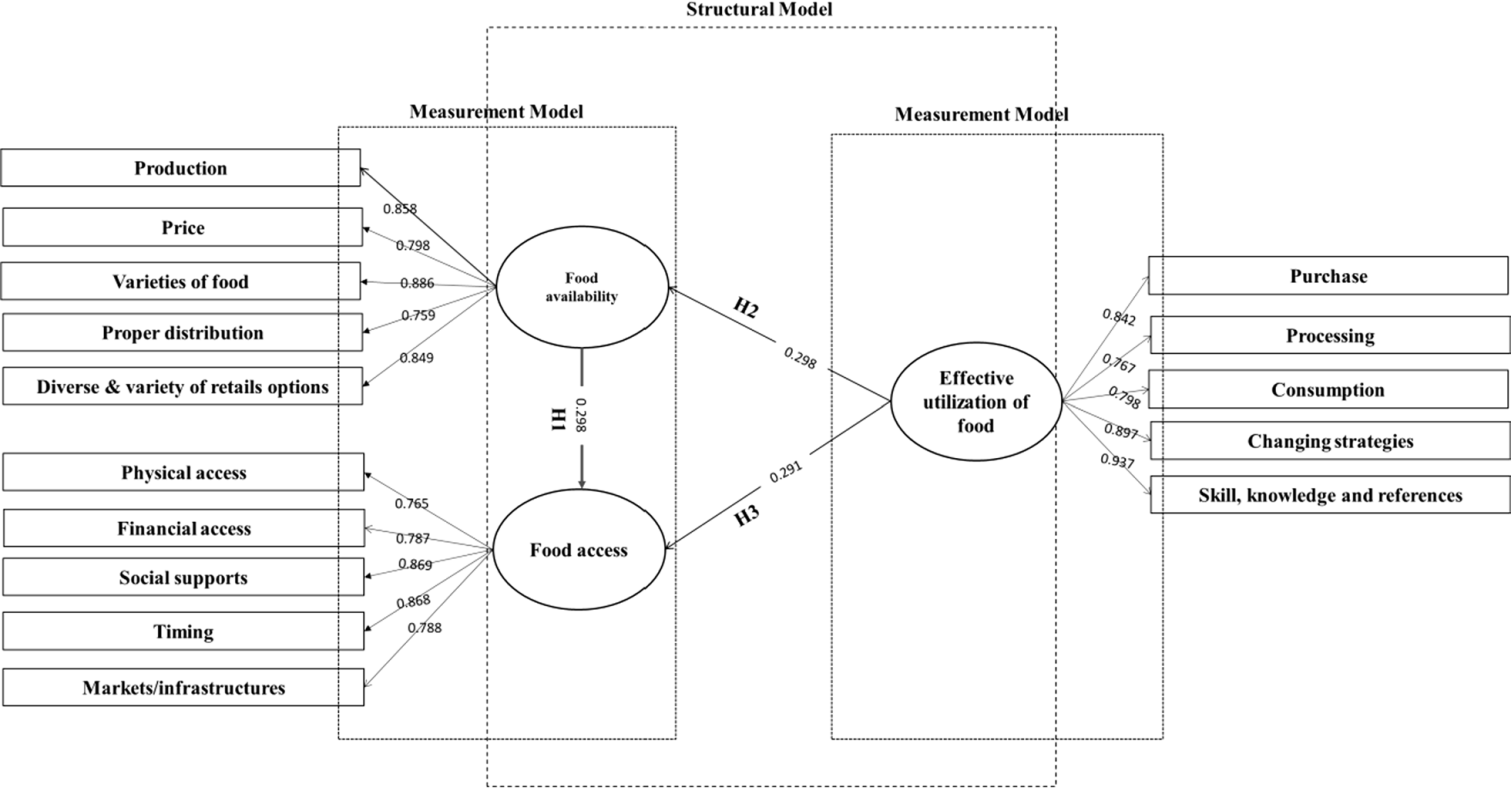
Complete SEM frameworks for the determinants of FS

Table 4 represents the findings of hypothesis testing. As *p* values were smaller than 0.05, and β values were positive for all three hypotheses. Therefore, the proposed framework represents a satisfactory level and is well-structured. The inner relationship among the determinants and the structural representation of the proposed framework are portrayed in Fig. 2.

**Table 4:** Results of the hypothesis test

	Hypothesis	β-value	*p* values	Results
*H1*	The “effective utilization of food” would be positively related to “food access”	0.291	0.029	Accepted
*H2*	The “effective utilization of food" would be positively related to "food availability”	0.298	0.011	Accepted
*H3*	The "food availability" would be positively related to "food access”	0.128	0.002	Accepted

The framework shows that the determinants for effective food utilization were positively related to the determinants of food availability and food access, and the determinants of food availability were positively related to the food access determinants.

## Discussion

Before COVID-19, persistent and severe starvation had risen due to many variables, including the holocaust, vulnerable social systems, environmental threats, global warming, and pesticides, decreased wages, and interrupted distribution networks. In this study, we studied the determinants of food security within three distinct aspects (effective utilization of food, food availability, and food access) within the COVID-19 epidemic situation. Within COVID-19, we had traced a higher impact between effective utilization of food and food availability and food access. Meanwhile, previous studies suggested that effective utilization of food has been triggered by changing behavior [61]. The concept of food security is broad and depends on several indicators that could have staggering impacts due to the consequences of the COVID-19 pandemic.

The significant findings indicated that respondents were having physical access to food, associated timing of getting food, and market structure or infrastructure, social networking (food access) were more importantly responsible for their food security as they had quantified significantly within the price, varieties, broad retail option, production, and distributions (food availability). Moreover, we had traced valuable impacts among the purchasing power, processing, habitual changes, and consumption patterns (effective utilization of food) within the aspects of food access. The personnel of food production and distribution industries must have proper access to food to maintain effective food utilization and therefore fostered the impacts of proper food production and distrusted within the national level [62]. Rozaki [28] indicated that policies that can enhance the food availability and smooth transition of food access, particularly, can increase the level of effective utilization of food and associated beneficial effects on securing the food within the vulnerable sectors like food production. The study found, smooth transition of food production, distribution, and available food retails option could trigger the proper utilization of food, which is parallel with the findings of Amjath-Babu et al. [63].

Several other studies have examined food availability and purchasing behavior [35]. While our research was designed to inquire specifically for food security inference within the special pandemic situation (COVID-19), the actions made in other aspects of their lives could have been applied towards their own compliance choice. Several factors could improve the agricultural sector’s welfare and food security in Shaanxi within the next few years, as COVID-19 may have possessed staggering threat towards national food security within the optimum level [64]. Our study further evinced the implication of a relatively complex land usage policy like China. In contrast, the production and distribution sectors are relatively vulnerable, especially in the context of infrastructure and sense of land entitlement, which is unexpected and could impede the agricultural sector from their access to legal rights, especially land rights. These are the central aspects for strengthening food security through enhanced negotiation capacity, increased farm production, and enhanced agricultural production and distribution [65]. In practices, it has been traced that the agricultural sectors invade freedom and support to maintain a smooth transition to provide effective utilization of food by availing food access and availability, despite heavy transition of lockdown and restrictions on movements [66].

Initiatives designed at increasing food security in the agriculture sector must incorporate local farmers’ and distributors’ insights and interests in implementing schemes customized to their needs. Local producers and distributors might contribute to skill development, recognize the proper usage of food within the community and allow scholars to learn about their domestic and customary structures [67]. Major infrastructure and communications improvements are required to motivate and resolve food insecurity issues for agricultural producers and distribution firms. The first move can be made by investing in logistics to build efficient and flexible marketplaces and logistics, enabling farmers to reach those markets through roads and modes of transportation. Strategic changing and flexible processing patterns should be encouraged to facilitate the effective utilization and eventually improve the stand towards maintaining the smooth availability of varieties of nutritious food. The authority must also consider providing more supports to maintain smooth transportation of agriculture products on a priority basis within these very tightly locked down scenarios to protect the small frame, agricultural workers, and value chains.

Our evaluation offered an overview of the different aspects of food security indicators within special pandemic situations triggered by many other existing externalities like ever-increasing climate change, land degradation, and global warming. As the access to food has been largely interpreted by the strict lockdown policy set by the government, in the study area, it is anticipated that the food availability should strongly correlate with food accessibility and be responsible for triggering the factors of effective food utilizations. This is aligned with the measurement approaches of core food security indicators [68] and managing food security within crisis moments [69].

Our findings have some policy implications. Decision-makers must keep working within the food production and distribution industries to predict, recognize, and resolve potential challenges. This critical situation arising from the COVID-19 outbreak could be a better opportunity for the government to plan or initiate the implementation of the long-awaited reformation of the existing agricultural product supply to the more digital paradigm. The study compiles the following recommendation for the policymaker for better transition of food security within the circumstances of COVID-19:

1. A transition towards digital farming and platforms will also be intensified, and Asian developing nations will have to deal with this changing setting to improve the agricultural sector’s productivity.
2. The changes will also reorient government and industry entities’ positions in terms of agricultural development, consumer safety, and the transportation systems of the distribution chain, quality management, and growth. Sufficient resources and profit should be allocated through agricultural modernization among the small-scale farmers and low-income agriculture populations.
3. Eventually, structural reforms will encourage new jobs opportunity, the competitiveness of the economy, governance, and food quality regulation to ensure food security for poor and small farmers.

To the best of our knowledge, the study will be the first attempt to identify, compile and structure the determinants of food security under the COVID-19 situation. Our study has some limitations as well. As the framework was developed by using the data gathered within the context of recent outbreaks of COVID-19 and in an emerging nation, there is a possibility of data heterogeneity. The studies focused on a specific area, and with a limited number of observations were gathered. The framework could be potential for further exploration with a wide range of sectors and scenarios. Moreover, if the framework can test within the contexts of different regions and endure agricultural food products, it would have been more interesting.

## Conclusions

This study constructed a statistically valid and reliable framework that quantified the determinants of FS within the circumstances of the COVID-19 outbreak. The 15 reliable and valid determinants of FS were extracted from an in-depth literature investigation from several published peer-reviewed journal articles, different books, and various reports, along with some discussion with professors and industry professionals and were categorized into three aspects (effective utilization of food, food availability, and food access) by using the SPSS statistical tool. The framework had been verified with the help of SEM tactics. Hypotheses encouraged a positive and substantial connection amongst the identified dimensions as those were evaluated with the help of the data set collected from the Chinese agricultural food production and distribution industries. The hypothesis test implied that the three aspects of food security mainly work in an integrated manner, which means they were interrelated. In other words, the effective utilization of food aspects was the root factor in this dynamic relationship as it connected with the other aspects. Moreover, from hypothesis testing, it could be seen that the accessibility aspects set dimensional effects within the food availability aspects. That means maintaining desirable food security; the interaction amongst determinants should be highlighted and carefully handled by the policymaker. The significance of this analysis is that it established the nexus within the determinant of FS, by which policymakers can make efficient use and leverage the root determinants for availing food security within local and global aspects. Seemingly, this study provided a recent theoretical overview of the determinants of FS with a special focus on food production and distribution industries within the COVID-19 epidemic circumstances.

## Supplementary Information

**Additional file 1.** The excerpt questionnaire of the study and Common Method Bias Test.

## Data Availability

The dataset used and analyzed during the current study is available from the Corresponding authors on reasonable request.
