# Supplementary material for: Evaluation of the determinants of food security within the COVID-19 pandemic circumstances- a particular case of Shaanxi, China
Source: Glob Health Res Policy. 2021 Dec 1;6:45. doi: 10.1186/s41256-021-00230-2 (PMC8632560; doi:10.1186/s41256-021-00230-2)
Supplement: Supplementary file 1 — Additional file 1. The excerpt questionnaire of the study and Common Method Bias Test. [file 41256_2021_230_MOESM1_ESM.docx]

**Additional file 1**

**Common Method Bias Test**

Since all of the survey elements were estimated with a specific form of the respondent (from food production and distribution industries) and a five-point Likert scale, a typical methodological bias may, therefore, exist throughout this analysis. Thereby, the effect of the common method bias has been sincerely evaluated. While there are just a limited number of present forecasts for determining the impact of common method bias (1), we adopted two approaches to defining common bias issues, i.e., Exploratory factor analysis and Harman’s single-factor test.

**Exploratory factor analysis**

Exploratory factor analysis (EFA) is a mainstream structured process that is required for evaluating the identified and endogenous factors at the interval or ratio level (2). In terms of the framework’s factor loadings, we assessed all the latent determinants, as shown in table 1. The early period of the assessment usually contains a wide range of provisional determinants. Thus factor rotation should be done for transforming the initial level factors for a better interpretation of results (3). For performing the principal components analysis, this study used the varimax rotation tactics. According to Mumtaz & Parahoo (4), for measuring inner precisions and Harman’s Single factor test and exploratory factor analysis (EFA) could be useful to measure a single measurement of the indicators for the rest of the mechanisms. The assessment of the measurement system could be quantified with the conceptual modeling to determine the interdependencies and interconnections among variables X (effective utilization of food), Y (food availability), and Z (food access). The evaluation furthermore verified that the items of the scales used were representative of the study. A satisfactory level of inner relationship (factor loadings) has been found among the determinants and their categories.

**Table S1 Exploratory factor analysis**

| Kaiser-Meyer-Olkin Measure of sampling adequacy |  | 0.797 |
| --- | --- | --- |
| Bartletts's test of sphericity | Approx. chi-square | 1023.256 |
|  | Df | 28 |
|  | Sig | 0.02 |

The Kaiser-Meyer Olkin (KMO) measure of the indicators has been found as 0.797, which denotes more than the required minimum values of 0.6 (5,6). Bartlett’s test of sphericity of significance (*P*=0.02) implies that the research could have been perceived by a credible measurement model with statistical importance of p<0.05, as suggested by Lindell & Whitney (5). The preliminary report (table 1) also implies that the indicators have Bartlett’s sphericity (χ²) score (1023.256), with a 28% degree of freedom and significance level of 0.020.

**Table S2 Descriptive statistics and factor analysis (within each factor)**

|  | Descriptive statistics of data | | | | |  | Factor loading | | |
| --- | --- | --- | --- | --- | --- | --- | --- | --- | --- |
| Determinants | Mean | SD | CITIC | SMC | CAID |  | Factor 1 | Factor 2 | Factor 3 |
| Price of food | 4.00 | 1.09 | 0.532 | 0.533 | 0.83 |  |  |  | 0.879 |
| Production | 3.64 | 1.06 | 0.698 | 0.490 | 0.82 |  |  |  | 0.755 |
| Varieties of food | 3.75 | 1.01 | 0.501 | 0.506 | 0.83 |  |  |  | 0.743 |
| Proper distribution channel | 3.78 | 1.06 | 0.589 | 0.554 | 0.83 |  |  |  | 0.716 |
| Diverse & variety of retails options | 3.76 | 1.00 | 0.503 | 0.430 | 0.81 |  |  |  | 0.734 |
| Purchase | 3.86 | 0.98 | 0.594 | 0.399 | 0.84 |  | 0.865 |  |  |
| Processing | 3.78 | 1.03 | 0.508 | 0.532 | 0.83 |  | 0.796 |  |  |
| Consumption | 3.63 | 1.02 | 0.530 | 0.675 | 0.84 |  | 0.854 |  |  |
| Changing strategies | 3.89 | 1.07 | 0.569 | 0.465 | 0.82 |  | 0.748 |  |  |
| Skill, knowledge and references | 3.61 | 1.01 | 0.635 | 0.598 | 0.82 |  | 0.856 |  |  |
| Physical access | 3.79 | 1.04 | 0.554 | 0.597 | 0.84 |  |  | 0.768 |  |
| financial access | 4.00 | 0.97 | 0.579 | 0.476 | 0.84 |  |  | 0.778 |  |
| Markets/infrastructures | 3.74 | 1.02 | 0.587 | 0.479 | 0.83 |  |  | 0.856 |  |
| Social supports | 2.97 | 1.09 | 0.579 | 0.529 | 0.82 |  |  | 0.756 |  |
| Timing | 3.87 | 0.98 | 0.564 | 0.456 | 0.84 |  |  | 0.890 |  |
| % of variance explained | | | | | | | 63.089 | 62.973 | 62.097 |
| Note: SD=standard deviation, CITIC=corrected item-total correlation, SMC=squared multiple correlation, CAID=Cronbach’s α if item deleted. | | | | | | | | | |

Corrected item-total correlation (CITC) denotes the correspondence of a component or factor measured with a composite score of the entire set of the remaining component within the same set. Table three proves that all the determinants a well ahead of the recommended CITIC value of 0.50 (7). Table three also shows that the determinants possessed a mean value within 3.61 to 4.0000 amongst the five scales. This implies that the observation considered the assessed determinates as a valuable assumption for securing food supply and security. By assessing Cronbach’s α-value, the reliability and viability of the determinants have been assured. Table three denotes CAID values for all the determinants that are well above the recommended CAID values, as stated by P. Barrett (8).

**Harman’s single-factor test**

Within the context of the study, we applied “Harman’s single-factor test,” as compiled by Podsakoff et al. (9) and Tehseen et al. (1). In this research, the principal component analysis (PCA) and unrevealed correlation matrix were employed with the entire latent construct included in this research to evaluate if a single factor arises or a single factor accounted for more than 50% of the co-variation. The measurements revealed that the overall variance accounted for 66.092% of the five factors from fifteen determinants, whether the first indicators hold with 49.028% variance within the dataset. Overall interpretation of table 2 shows that the dataset does not contain any single factor bias issues, and the first factors’ values also did not lead with the most variance. After concluding all the preliminary assessments of table one, two, and three, it can be confidently stated that all the collected data are viable and valid for further analytical approaches.

**Table S3 Harman’s single factor test**

| Component | Initial Eigenvalues | | |  | Extraction Sums of Squared Loadings | | |
| --- | --- | --- | --- | --- | --- | --- | --- |
|  | Total | % of variance | Cumulative % |  | Total | % of variance | Cumulative % |
| Price of food | 14.866 | 49.028 | 49.028 |  | 14.866 | 49.028 | 49.028 |
| Production | 1.589 | 5.451 | 54.479 |  | 1.589 | 5.451 | 54.479 |
| Varieties of food | 1.468 | 4.576 | 59.055 |  | 1.468 | 4.576 | 59.055 |
| Proper distribution channel | 1.085 | 3.621 | 62.676 |  | 1.085 | 3.621 | 62.676 |
| Diverse & variety of retails options | 0.986 | 3.416 | 66.092 |  | 0.986 | 3.416 | 66.092 |
| Purchase | 0.897 | 3.073 | 69.165 |  |  |  |  |
| Processing | 0.765 | 2.518 | 71.683 |  |  |  |  |
| Consumption | 0.629 | 2.142 | 73.825 |  |  |  |  |
| Changing strategies | 0.576 | 1.956 | 75.781 |  |  |  |  |
| Skill, knowledge, and references | 0.546 | 1.864 | 77.645 |  |  |  |  |
| Physical access | 0.536 | 1.871 | 79.516 |  |  |  |  |
| financial access | 0.523 | 1.787 | 81.303 |  |  |  |  |
| Markets/infrastructures | 0.501 | 1.789 | 83.092 |  |  |  |  |
| Social supports | 0.596 | 1.853 | 84.945 |  |  |  |  |
| Timing | 0.580 | 1.876 | 86.821 |  |  |  |  |

**References:**

1. Tehseen S, Sajilan S, Gadar K, Ramayah T. Assessing cultural orientation as a reflective-formative second order construct-a recent PLS-SEM approach. Review of Integrative Business and Economics Research. 2017;6(2):38.

2. Austin JT, Calderón RF. Theoretical and technical contributions to structural equation modeling: An updated annotated bibliography. 1996;

3. Kumar Mittal V, Singh Sangwan K. Development of a structural model of environmentally conscious manufacturing drivers. Journal of Manufacturing Technology Management. 2014 Jan 1;25(8):1195–208.

4. Mumtaz S, Parahoo SK. Promoting employee innovation performance. International Journal of Productivity and Performance Management. 2019;

5. Lindell MK, Whitney DJ. Accounting for common method variance in cross-sectional research designs. Journal of applied psychology. 2001;86(1):114.

6. Torabizadeh M, Yusof NM, Ma’aram A, Shaharoun AM. Identifying sustainable warehouse management system indicators and proposing new weighting method. Journal of Cleaner Production. 2020;248:119190.

7. Koufteros XA. Testing a model of pull production: a paradigm for manufacturing research using structural equation modeling. Journal of operations Management. 1999;17(4):467–88.

8. Barrett P. Structural equation modelling: Adjudging model fit. Personality and Individual Differences. 2007 May 1;42(5):815–24.

9. Podsakoff PM, MacKenzie SB, Lee J-Y, Podsakoff NP. Common method biases in behavioral research: a critical review of the literature and recommended remedies. Journal of applied psychology. 2003;88(5):879.
